# Polyphenols and Neurodegenerative Diseases: Knowledge-Mining Insights, Mechanistic Evidence, and Emerging Nutritional Applications

**DOI:** 10.3390/nu18040602

**Published:** 2026-02-12

**Authors:** Xiaomei Wang, Huimin Zhao, Jiao Yang, Jiayuan Zhang, Yiqin Zhang, Jian Zhu, Mei Mei, Gaihong Yu, Guojian Xian, Ruixue Zhao, Yingli Nie

**Affiliations:** 1 Institute of Agricultural Information, Chinese Academy of Agricultural Sciences, Beijing 100081, China; 2 College of Food Science, Sichuan Agricultural University, Ya’an 625014, China; 3 Institute of Medical Information, Chinese Academy of Medical Sciences, Beijing 100190, China; 4 National Science Library, Chinese Academy of Sciences, Beijing 100020, China

**Keywords:** polyphenols, neurodegenerative diseases, knowledge-mining, mechanisms, hotspots

## Abstract

Polyphenols are a diverse group of plant-derived bioactives that have been investigated as multi-target candidates for the potential prevention and management of neurodegenerative diseases (NDDs). We conducted an integrated bibliometric and mechanistic scoping review covering 12 polyphenol classes and seven major NDDs using records from PubMed, Embase, the Cochrane Library, and Web of Science (1940–2024). Research landscapes and emerging themes were mapped using keyword co-occurrence, clustering analyses, and BERTopic modeling. Mechanistic evidence was synthesized across core pathways, including oxidative stress, neuroinflammation, proteostasis (amyloid/tau and α-synuclein), mitochondrial dysfunction, and cholinergic modulation, to link preclinical findings with clinical outcomes. Publication output increased markedly after 2000, with China and the United States contributing the most records. Four persistent hotspots were identified: (1) antioxidant and neuroprotective effects (e.g., resveratrol, curcumin); (2) anti-inflammatory activity and intracellular signaling; (3) cognition, aging, and sex-specific responses in clinical research; and (4) animal models of memory impairment. Clinically investigated interventions include epigallocatechin gallate, Ginkgo biloba extracts, olive/cocoa polyphenols, and flavonoid-rich mixtures; however, limited bioavailability and heterogeneous trial designs constrain the strength of effect estimates. Advances in delivery systems, computational screening, and precision nutrition may improve translation. Overall, polyphenols show multi-target neuroprotective potential, but larger and more standardized clinical trials are needed to support evidence-based nutritional strategies for NDD prevention and management.

## 1. Introduction

Neurodegenerative diseases (NDDs), including Alzheimer’s disease (AD) and Parkinson’s disease (PD), represent a pressing global health challenge, characterized by progressive cognitive decline and loss of neuronal integrity. With aging populations worldwide, the prevalence of these disorders is rapidly increasing, placing an immense burden on healthcare systems and society [1]. For example, dementia affected 57 million people worldwide in 2021, with nearly 10 million new cases annually, and cost US$1.3 trillion in 2019, while PD affected over 8.5 million people globally in 2019, causing 5.8 million disability-adjusted life years and approximately 329,000 deaths [2,3]. While pharmacological treatments exist, they often provide limited efficacy in halting disease progression, underscoring the urgent need for alternative, preventive, and therapeutic strategies.

Diet and nutrition have emerged as pivotal factors in promoting brain health and mitigating the risks of NDDs. Among dietary components, polyphenols—a diverse group of plant-derived bioactive compounds—have attracted considerable interest for their neuroprotective potential. Found abundantly in fruits, vegetables, tea, and wine, polyphenols are recognized for their potent antioxidant, anti-inflammatory, and neuro-modulatory properties [4,5,6]. Preclinical studies have suggested that polyphenols may counteract oxidative stress, reduce neuroinflammation, and enhance synaptic plasticity, all of which are implicated in the pathogenesis of NDDs [7,8]. Notably, dietary patterns rich in polyphenols, such as the Mediterranean diet, have been associated with a reduced incidence of cognitive decline and dementia [9].

Recent studies have substantially advanced our understanding of how polyphenols may modulate NDD-related pathophysiology. Across preclinical models, polyphenols have been reported to attenuate oxidative stress and neuroinflammation, regulate neurotrophic signaling (e.g., BDNF-related pathways) [10], and interfere with pathological protein aggregation, including β-amyloid and α-synuclein [11,12]. In addition, accumulating animal and human studies suggest potential benefits on cognitive outcomes and disease-related symptoms, although the magnitude and robustness of effects vary across compounds, formulations, and study designs. For example, epigallocatechin gallate (EGCG) and Ginkgo biloba extracts have been evaluated in clinical settings with mixed but promising signals [10,13].

Meanwhile, the literature has expanded rapidly across multiple research directions, including structure–activity relationships, oxidative stress biology, animal models, molecular docking, and formulation development. However, most existing reviews focus on selected compounds, pathways, or single diseases (most commonly Alzheimer’s disease) [14,15,16] and therefore provide limited quantitative insight into how the field has evolved over time (e.g., growth phases, geographic leadership, collaboration networks, and emerging themes). Bibliometric mapping can complement narrative synthesis by objectively identifying influential contributors, knowledge clusters, and temporal bursts of attention [17,18]. Accordingly, this scoping review integrates bibliometric analyses with a mechanism-oriented synthesis to (i) map the global research landscape across 12 polyphenol classes and seven major NDDs (1940–2024), (ii) identify hotspots and topic evolution using co-occurrence/clustering and BERTopic modeling, and (iii) connect bibliometric patterns with mechanistic and translational evidence to highlight knowledge gaps and future directions.

## 2. Methodology

### 2.1. Scoping Review

**Figure 1:**
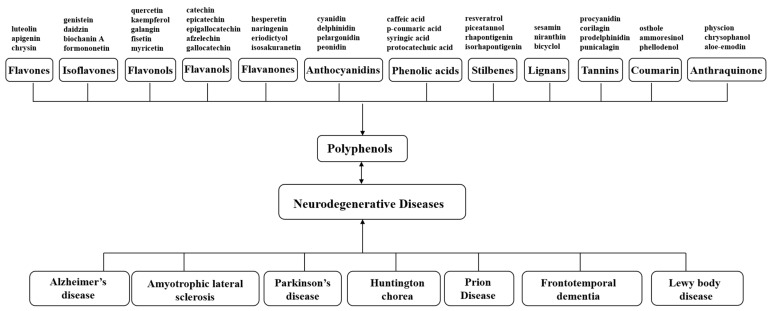
Classification framework of 12 polyphenol types and 7 major neurodegenerative diseases. Schematic overview of the study scope, showing the 12 predefined polyphenol classes (flavones, isoflavones, flavonols, flavanols, flavanones, anthocyanidins, phenolic acids, stilbenes, lignans, tannins, coumarins, and anthraquinones) and the seven target neurodegenerative diseases (Alzheimer’s disease, Parkinson’s disease, Huntington’s disease, amyotrophic lateral sclerosis, frontotemporal dementia, prion disease, and Lewy body dementia). The framework guided search-term construction, record tagging (polyphenol class and disease labels), and subsequent bibliometric mapping and mechanism-oriented synthesis.

Guided by the PRISMA Extension for Scoping Reviews (PRISMA-ScR), we conducted a scoping review to map the research landscape, hotspots, and translational potential of polyphenols in neurodegenerative diseases (NDDs) by integrating bibliometric analyses with mechanistic evidence synthesis. Using these guidelines, we developed a protocol. We identified and categorized 12 polyphenol classes, including flavones, isoflavones, flavonols, flavanols, flavanones, anthocyanidins, phenolic acids, stilbenes, lignans, tannins, coumarins, and anthraquinones, to understand their specific biological activities and potential effects on 7 clinically important and commonly studied NDDs, such as Alzheimer’s disease, Parkinson’s disease, Huntington’s syndrome, amyotrophic lateral sclerosis, frontotemporal dementia, prion disease, and Lewy body dementia (Figure 1). The methodology integrates bibliometric analysis and clustering techniques to derive insights from diverse datasets.

A structured search strategy was developed using a predefined concept framework covering polyphenol classes and major neurodegenerative diseases (NDDs). We first compiled candidate controlled-vocabulary terms and free-text synonyms for each concept based on pilot searches and iterative refinement. We then constructed database-specific executable queries by combining polyphenol terms and NDD terms using Boolean operators (AND/OR/NOT) and parentheses, with platform-specific field tags and syntax (e.g., MeSH terms and tiab fields in PubMed; Emtree terms and database field tags in Embase; TS= and proximity operators where supported in Web of Science; and Cochrane Library syntax including MeSH descriptors and title/abstract/keyword fields, as applicable). Controlled-vocabulary handling (including explode/non-explode settings where available), all limits/filters (e.g., English language; coverage up to 31 December 2024), and the final retrieved record counts are reported. Complete database-specific search strings and corresponding results are provided in Supplementary File S1 to ensure reproducibility.

### 2.2. Data Collection and Preparation

#### 2.2.1. Search Strategy Construction

A structured search strategy was developed based on the defined search terms and logical framework. Searches were built using keywords and Boolean operators related to polyphenols and neurodegenerative diseases (NDDs). The search terms were customized for each database to ensure comprehensive coverage. Alongside the creation of the search query, a pilot search was conducted with the aim of identifying relevant keywords. The definition of the search query was an iterative process, where the output of 1 search query was used to alter the subsequent search query. Specifically, the search terms were determined according to the keywords illustrated in Figure 1, including both subject headings (e.g., MeSH terms in PubMed, Emtree terms in Embase) and free-text terms. The final strategies were tailored to the syntax and logical operators of each database, thereby ensuring both sensitivity and specificity in retrieving relevant literature. Database-specific full search strings (including controlled-vocabulary terms and free-text terms, with all filters/limits) are reported in Supplementary File S1.

#### 2.2.2. Eligibility Criteria and Study Selection

We predefined eligibility criteria consistent with a scoping review objective. Records were included if they: (1) were retrieved from PubMed, Embase, Cochrane Library, or Web of Science; (2) addressed polyphenols (including any of the 12 predefined polyphenol classes and/or representative compounds) in relation to at least one of the seven target NDDs; and (3) reported relevant outcomes or mechanistic information (preclinical, clinical, or translational) or contributed bibliometric metadata suitable for mapping analyses.

**Figure 2:**
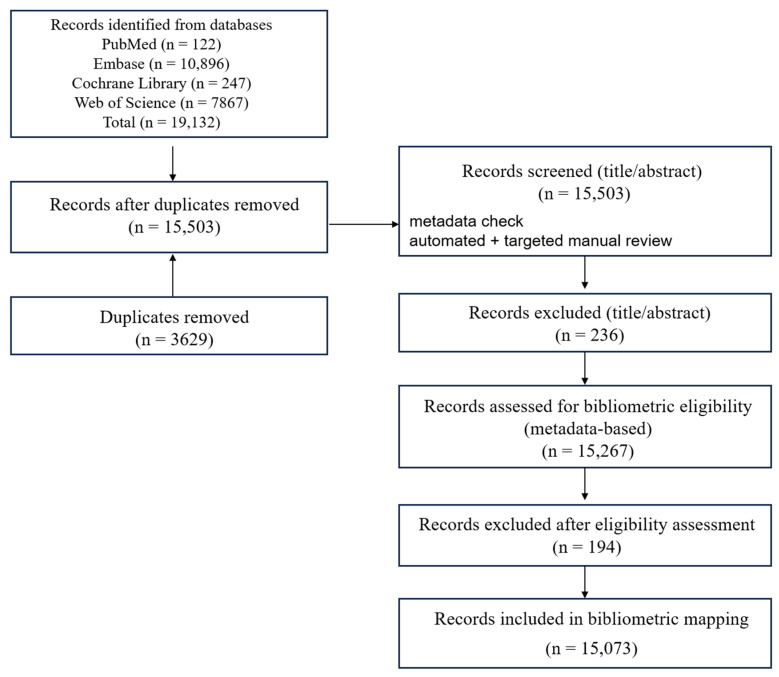
PRISMA-ScR flow diagram of study identification, screening, eligibility assessment, and inclusion for the bibliometric scoping review. Flow diagram summarizing record identification across PubMed, Embase, Web of Science Core Collection, and the Cochrane Library, followed by de-duplication, title/abstract screening, metadata-based eligibility assessment, and final inclusion in the bibliometric dataset. Numbers at each step indicate records retained/excluded, with reasons for exclusion reported according to PRISMA-ScR guidance.

Records were excluded if they: (1) were duplicates; (2) were not relevant to polyphenols and NDDs after title/abstract screening; (3) were non-scholarly publication types (e.g., editorials, letters, corrections, meeting abstracts without sufficient metadata); or (4) lacked sufficient bibliographic information required for bibliometric analyses. Study selection was performed by screening titles/abstracts against these criteria, followed by full-text assessment when needed for mechanistic synthesis. A PRISMA-ScR flow diagram and checklist are provided (Figure 2).

#### 2.2.3. Data Collection

Records were retrieved from four major databases: Web of Science (WoS), PubMed, Embase and Cochrane Library, focusing on research published up to 31 December 2024. This selection was based on expert knowledge in the field of nutrition and data science. The automated search was performed using a search query. Search queries based on the constructed strategy were executed in each of the selected databases to gather relevant studies and research articles. This ensures a broad collection of data across various platforms. Next to the automated search through the previously described libraries, a manual search was executed. The manual search was performed by going through the references of the articles that were found by the search string. We looked at both the sources the authors referenced and the ones they were referenced by and included all relevant articles. All database searches were executed from January 2025 to March 2025. The search was limited to English only. We included database-indexed scholarly record types with sufficient bibliographic metadata (e.g., articles/reviews and, where available, proceedings/early-access items, Cochrane reviews/protocols and CENTRAL trial records), while excluding conference abstracts and other non-scholarly items.

#### 2.2.4. Data Processing

Records retrieved from each database were exported in their native bibliographic formats (e.g., RIS/BibTeX/CSV/XML) and imported into EndNote to harmonize bibliographic fields and to append structured labels indicating polyphenol class and neurodegenerative disease (NDD) category. All records were then exported from EndNote in a unified format. Exports retained core fields required for bibliometric mapping (title, authors, affiliations, year, abstract when available, polyphenolic type label, disease type label, keywords/index terms, DOI/PMID, document type, and source database). Specifically, we (i) normalized field names across sources, (ii) unified date formats, (iii) standardized author, journal, and institutional names, (iv) harmonized keyword fields (including author keywords and controlled-vocabulary terms where available), and (v) tagged each record with its source database to preserve provenance. After harmonization, records were merged and assigned unique internal identifiers to enable traceability. To enable downstream processing in Derwent Data Analyzer (DDA), we used custom Python 3.9 (64-bit) scripts (provided as Supplementary File S2) to convert the EndNote export into a DDA-readable schema.

De-duplication and record merging were conducted using Derwent Data Analyzer (DDA, Clarivate^®^) built-in matching and merge rules. First, automatic de-duplication was performed via exact matching on unique identifiers available in the records, including ISSN, Web of Science Accession Number, and PMID. Second, remaining candidate duplicates were reconciled via a manual DOI-based check and merge, where DOI equality was used as the primary criterion. For records lacking stable identifiers or with suspected title-level duplication, we applied title-based screening to flag potential duplicates and then verified them using bibliographic metadata—abstract, specifically author(s), publication year, and journal/source title—before final merge decisions were made. All candidate duplicates flagged by DDA were manually reviewed by two independent reviewers; discrepancies were resolved by discussion and, when needed, adjudicated by a third senior reviewer. The final merged dataset retained one canonical record per duplicate set and preserved source provenance tags to enable traceability. In total, 3629 duplicates were removed, yielding 15,503 unique records. A limitation of this workflow is that we did not quantify a formal de-duplication error rate (e.g., false-merge or missed-duplicate rates). However, we prioritized deterministic identifier matching and metadata verification to reduce these risks and to support traceability.

Given the scale of the corpus, screening was performed using titles/abstracts and structured topical fields (keywords/index terms) rather than universal full-text retrieval. The 15,503 unique records were screened at the title/abstract level against predefined eligibility criteria, resulting in the exclusion of 236 records. The remaining 15,267 records underwent a metadata-based eligibility assessment for inclusion in the bibliometric mapping corpus, drawing primarily on bibliographic fields and topical information (year and/or author and/or institution/index terms). During this step, 194 records were excluded with documented reasons (e.g., out of scope upon closer inspection, non-eligible publication types, or insufficient metadata for bibliometric mapping and topic modeling). Ultimately, 15,073 records were included in the final bibliometric dataset (Figure 2). Full texts were consulted selectively only when needed to resolve ambiguous cases and to support targeted mechanistic/clinical evidence extraction.

#### 2.2.5. Dataset Construction

Following data merging, harmonization, de-duplication, and assessment, we obtained a refined and comprehensive dataset comprising standardized metadata fields essential for bibliometric analysis. The cleaned dataset systematically organizes key bibliographic elements, including complete study titles, normalized author lists with institutional affiliations, standardized publication years, harmonized keyword sets, and full-text abstracts when available. This structured dataset, free from redundancies and formatting inconsistencies, serves as the foundational corpus for all subsequent analytical operations.

### 2.3. Data Analysis

#### 2.3.1. Bibliometric Analysis

Publication trends, author collaborations, and institutional contributions were analyzed using the Derwent Data Analyzer (DDA) version 12.6 tool by Clarivate^®^. DDA was used to quantify annual publication outputs over time, summarize contributions by countries/regions and institutions, and generate collaboration maps (e.g., co-authorship and institutional collaboration networks). Standard bibliometric indicators such as publication counts and network-based collaboration structures were extracted to characterize the overall research landscape and its evolution.

#### 2.3.2. Clustering and Topic Modeling

The research methodology employs a combination of advanced analytical tools to identify emerging research trends and hotspots. First, VOSviewer version 1.6.10 is used to perform keyword clustering analysis on the entire dataset of literature, grouping related terms to identify key areas of research. Then, CiteSpace 6.3 R1 is applied to analyze the keywords from literature published in the last decade (2015–2024), conducting keyword clustering, co-occurrence analysis, time-zone analysis, and burst detection to explore the evolution of research topics, identify shifts in focus, and detect emerging trends over time. Finally, BERTopic is utilized to cluster and analyze the abstracts of all the literature, applying topic modeling and similarity analysis to reveal key research themes and their relationships. The integrated approach allows for a comprehensive identification of research hotspots and offers a deep understanding of the evolving research landscape.

#### 2.3.3. Study Type Classification

To support cautious translational interpretation, records were tagged as clinical or non-clinical using available metadata (e.g., publication type where provided by databases) and text cues in titles/abstracts (e.g., randomized, placebo-controlled, double-blind, patients, trial). This tagging was used only for narrative interpretation of translational relevance; no efficacy weighting, risk-of-bias weighting, or quantitative pooling was performed, consistent with a PRISMA-ScR scoping review and bibliometric mapping design.

## 3. Fundamentals of Polyphenols and Neurodegeneration

### 3.1. Definition and Classification of Polyphenols

Polyphenols are a diverse group of naturally occurring secondary metabolites in plants, characterized by multiple phenolic structural units. They are broadly classified into flavonoids (including flavones, isoflavones, flavonols, flavanols, flavanones, anthocyanidins), phenolic acids (e.g., caffeic acid, chlorogenic acid), stilbenes (e.g., resveratrol), lignans, tannins [19], and others (e.g., coumarins, anthraquinones). These compounds are abundant in fruits, vegetables, cereals, tea, coffee, and wine. Their structural heterogeneity confers a wide range of bioactivities, which makes them central candidates in the exploration of diet–disease interactions [20,21].

### 3.2. Mechanisms of Polyphenol Action in the Nervous System

Polyphenols have been reported to influence neurobiological processes through multiple, often interconnected, mechanisms [22]: Antioxidant defense: Many polyphenols act as free radical scavengers and upregulate endogenous antioxidant enzymes (e.g., superoxide dismutase, catalase, glutathione peroxidase), potentially contributing to reduced oxidative stress [23]. Anti-inflammatory regulation: They modulate neuroinflammatory pathways by inhibiting NF-κB signaling, downregulating pro-inflammatory cytokines (TNF-α, IL-6), and regulating microglial activation [24]. Amyloid and protein aggregation control: Certain compounds, such as resveratrol and epigallocatechin gallate (EGCG), influence amyloid precursor protein (APP) processing, inhibit β-amyloid aggregation, and reduce tau hyperphosphorylation [25,26,27]. Mitochondrial function and energy metabolism: Polyphenols enhance mitochondrial biogenesis and stabilize membrane potential, which is important for neuronal survival [28]. Neurotransmitter modulation: Some polyphenols inhibit acetylcholinesterase (AChE) and butyrylcholinesterase (BChE), prolonging acetylcholine activity, which may contribute to cognitive function [29].

### 3.3. Neuropathological Basis of Neurodegenerative Diseases

Neurodegenerative diseases such as Alzheimer’s disease (AD), Parkinson’s disease (PD), amyotrophic lateral sclerosis (ALS), Huntington’s syndrome (HS), Frontotemporal dementia (FTD), Lewy body dementia (LBD) and Prion disease share several overlapping pathological hallmarks: Protein misfolding and aggregation: Accumulation of β-amyloid plaques and tau tangles in AD, α-synuclein aggregates in PD, and mutant huntingtin in HD disrupt neuronal function. Oxidative stress and mitochondrial dysfunction: Excess reactive oxygen species (ROS) generation damages neuronal DNA, proteins, and lipids, accelerating disease progression [30]. Chronic neuroinflammation: Sustained activation of glial cells contributes to neuronal injury and synaptic loss. Excitotoxicity and calcium imbalance: Excessive glutamate release leads to calcium overload and neuronal apoptosis. Vascular dysfunction and metabolic impairment: Altered blood–brain barrier permeability and reduced cerebral glucose utilization further exacerbate cognitive decline [31]. Understanding these mechanisms is crucial, as they provide the molecular targets through which polyphenols exert their modulatory and protective effects. By linking polyphenol activity to specific neuropathological features, researchers can better elucidate their potential in disease prevention and therapy.

## 4. Current Advances in Research

### 4.1. Publication Trends and Global Distribution

**Figure 3:**
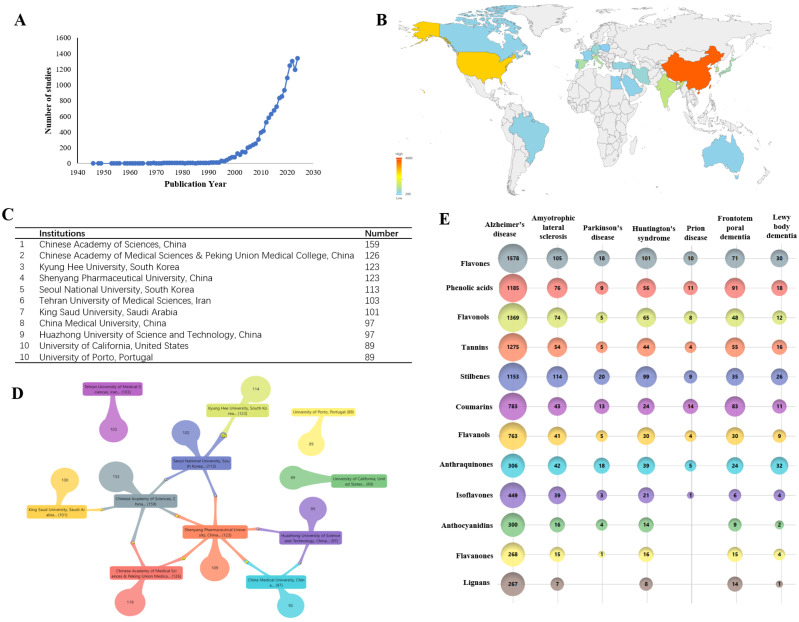
Global research output and distribution analysis. (**A**) Annual publication output on polyphenols and neurodegenerative diseases from 1940 to 2024. (**B**) Country/region contributions ranked by publication count in the final dataset. (**C**) Most productive institutions based on publication output. (**D**) Collaboration network (e.g., country or institutional co-authorship), where node size reflects publication volume and link thickness reflects collaboration strength. (**E**) Compound–disease association matrix summarizing the distribution of publications across polyphenol classes and neurodegenerative diseases in the dataset.

The longitudinal analysis of publication trends (Figure 3A) reveals consistent growth in research output from the 1940s through the 2020s, with particularly accelerated growth emerging in the 1990s as research interest in this field intensified globally. The sharp increase after 2000 is likely to reflect growing recognition of shared NDD mechanisms (oxidative stress and neuroinflammation), expansion of nutrition neuroscience/functional food research, advances in analytical and omics/biomarker methods, and intensified translational interest driven by population aging and dementia burden. The publication volume reached its zenith in 2024, recording an annual output of 1343 peer-reviewed publications, reflecting heightened scientific attention during this period.

Geographic distribution analysis demonstrates clear regional leadership, with China, the United States, and India collectively accounting for the majority of publications, while other scientifically active nations, including South Korea, Italy, and Japan, maintain substantial but comparatively smaller research outputs (Figure 3B). At the institutional level, the Chinese Academy of Sciences emerges as the most prolific contributor with 159 publications, closely followed by the Chinese Academy of Medical Sciences & Peking Union Medical College (126 publications). China’s high output likely reflects sustained investment and expanded research capacity in life sciences and nutrition/phytochemical research since the early 2000s; however, publication volume indicates research activity rather than evidentiary strength or clinical certainty.

Other notable contributing institutions span multiple continents, with significant representation from South Korean universities, Iranian research centers, and Saudi Arabian academic institutions (Figure 3C), illustrating the truly international character of this research domain. Network analysis further reveals established collaborative patterns, particularly among research institutions in China, South Korea, and Saudi Arabia (Figure 3D), suggesting the formation of regional research consortia focused on polyphenol–neurodegeneration studies.

The aggregated dataset comprises a total of 15,073 scientific records, encompassing studies investigating various neurodegenerative conditions and their associated bioactive compounds (Figure 3E). Disease-specific analysis reveals a strongly AD-centric research landscape linking phytochemical classes to major neurodegenerative disorders. Across nearly all compound categories, Alzheimer’s disease (AD) shows the largest publication volume, with particularly strong representation for flavones (1578), flavonols (1369), tannins (1275), phenolic acids (1185), and stilbenes (1153). Several other classes also display substantial AD coverage, including coumarins (783), flavanols (763), isoflavones (449), and anthraquinones (306), reinforcing AD as the primary disease focus in this literature. In contrast, other neurodegenerative conditions receive markedly fewer studies. ALS exhibits moderate counts across multiple compound types, while FTD and LBD show selective but noticeable interest. PD and HS generally remain low across most phytochemical groups, with only sporadic higher values. Prion disease is consistently the least studied category, with counts typically in the single digits to low teens.

### 4.2. Research Hotspots

**Figure 4:**
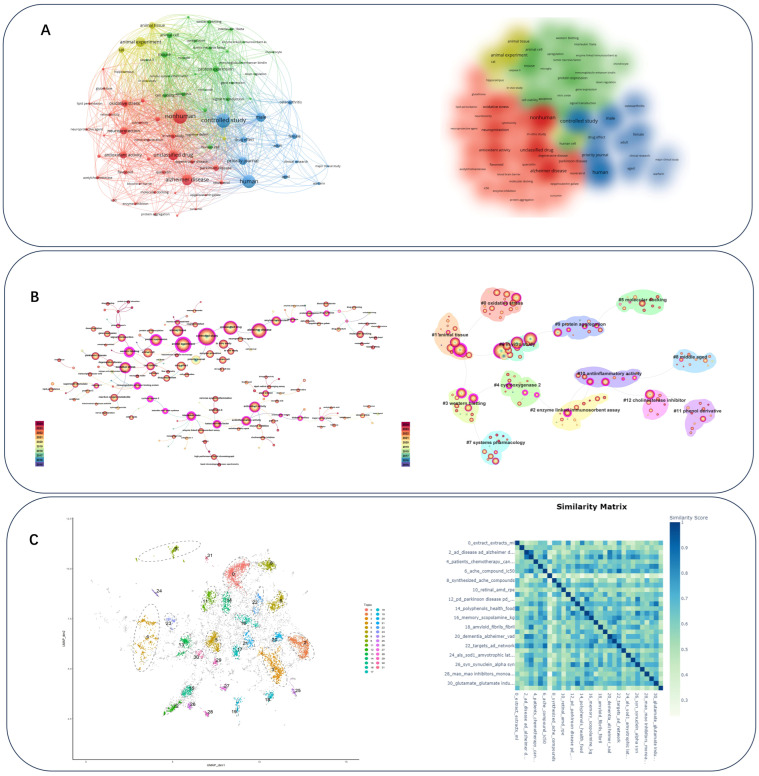
Research hotspots. (**A**) Keyword co-occurrence clustering map generated in VOSviewer, where nodes represent keywords, node size reflects frequency, and links indicate co-occurrence strength; colors denote clusters (research hotspots). (**B**) CiteSpace keyword clustering (13 clusters) for publications from 2015 to 2024, illustrating major thematic areas and their interconnections; labels represent the dominant terms within each cluster. (**C**) BERTopic modeling of abstracts, showing the main latent topics identified across the full corpus and their relationships/similarities.

The cluster analysis of 15,073 keywords highlights key research hotspots across four categories (Figure 4A). Category 1 focuses on neuroprotection and oxidative stress, with studies investigating antioxidant-rich plant extracts (like resveratrol and curcumin) for their potential in treating neurodegenerative diseases such as Alzheimer’s and Parkinson’s. This research emphasizes enzyme activity, neurotoxicity, and methods like molecular docking for drug discovery. Category 2 explores inflammation and cellular mechanisms, focusing on neuroinflammation and its impact on neurodegenerative diseases through techniques like Western blotting and ELISA to analyze protein expression, apoptosis, and gene expression in response to inflammatory mediators. Category 3 emphasizes clinical research, particularly drug effects on aging populations and gender differences in drug responses, with a focus on clinical trials for diseases like neurodegenerative disorders. Category 4 examines animal models to study cognitive function, using rats and mice to explore the effects of compounds on memory impairments and cognitive decline, key in developing treatments for Alzheimer’s. In summary, the hotspots highlight major research foci in the field, spanning efficacy-oriented experiments, cellular mechanisms, and animal model research.

CiteSpace conducted a keyword clustering analysis on 12,000 data points from research published since 2015, resulting in 13 clusters representing key research areas (Figure 4B). The 13 keyword clusters identified through CiteSpace analysis represent distinct research themes across various scientific fields. Cluster 1 focuses on oxidative stress and neurodegenerative diseases, with keywords like superoxide dismutase and glutathione. Cluster 2 centers on animal models and experimental research, particularly in relation to cognitive testing and neurodegeneration. Cluster 3 explores flavonoids and their role in vascular health and diabetes. Cluster 4 delves into immune responses, while Cluster 5 connects neurotrophic factors with inflammation and drug development. Cluster 6 emphasizes molecular docking and drug discovery techniques. Clusters 7 through 13 focus on Parkinson’s disease, neuroprotection, cholinergic transmission, antioxidant activity, clinical trials, and protein aggregation, with an emphasis on experimental and biomolecular studies.

Abstracts were analyzed using BERTopic to identify thematic clusters (Figure 4C). The research topics in polyphenol–neurodegeneration studies, derived from KeyBERT and MMR, are presented in Table 1. BERTopic modeling of abstracts yielded themes that can be consolidated into six overarching domains, each defined by coherent mechanistic or translational emphases and supported by the topic-similarity structure. (1) Dietary sources and polyphenol-related materials focus on plant extracts, functional foods, and representative dietary polyphenols (Topics 0, 14, 19). (2) Alzheimer’s disease-centered pathology and cognition capture AD/dementia and cognitive outcomes together with core proteinopathy processes, including Aβ/amyloid formation and tau/tauopathies (Topics 2, 3, 18, 20, 21, 25). (3) Parkinson’s disease and synucleinopathies cover dopaminergic dysfunction, MPTP-related experimental paradigms, and α-synuclein aggregation (Topics 12, 15, 26). (4) Shared mechanistic modules across NDDs emphasize oxidative stress, microglia-driven neuroinflammation, mitochondrial dysfunction, and glutamate excitotoxicity (Topics 5, 13, 29, 30). (5) Pharmacological targets and intervention contexts include cholinergic targets (AChE inhibitors and related synthesized compounds), MAO-related mechanisms, estrogen-related themes, and patient/drug contexts (Topics 6, 8, 28, 27, 4). (6) Other NDDs and translation-enabling strategies highlight prion disease, ALS/SOD1-related work, and brain-directed delivery technologies such as nanoparticles (Topics 9, 24, 23).

### 4.3. Topic Evolution

**Figure 5:**
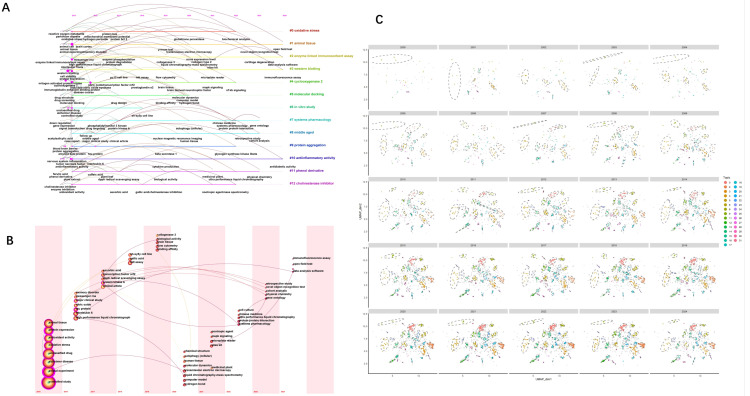
Temporal evolution of research themes. (**A**) CiteSpace keyword timeline view (2015–2024) showing the emergence and persistence of major clusters over time. (**B**) Keyword time-zone visualization highlighting shifts in thematic emphasis across years; earlier versus later nodes reflect topic progression and turning points in the field. (**C**) Topic overlap dynamics (2000–2024) illustrating changes in topic connectivity and interdisciplinarity over time, with increased overlap indicating convergence of mechanistic, translational, and methodological themes.

The 13 keyword timelines show the evolution of these research areas over time (Figure 5A). In 2016, research on oxidative stress and neurodegenerative diseases gained prominence, with superoxide dismutase and related markers being central. By 2017, animal models and behavioral research were a focus, alongside flavonoids and their impact on vascular and metabolic diseases. 2018 saw the rise in research into neurotrophic factors, cholinergic transmission, and the potential of plant-based compounds like chlorogenic acid. The timeline also highlights growing interest in drug discovery and molecular modeling, with clinical trials on Parkinson’s disease. Throughout the analysis, centrality values indicate the increasing significance of these keywords, reflecting trends and shifts in scientific focus from experimental studies to clinical applications and therapeutics.

The keyword time zone chart summarizes how research priorities evolved from 2015 to 2024 (Figure 5B). Early work (2015) focused on foundational mechanisms such as oxidative stress and drug action, largely using protein expression and in vitro approaches. From 2016 to 2018, attention expanded to molecular docking, immune/neuroinflammatory responses, and neuroprotective bioactives (e.g., flavonoids). Between 2019 and 2020, clinical translation became more prominent, emphasizing bioavailability, biomarkers, and therapeutic interventions. From 2021 onward, keywords increasingly reflected clinical outcomes, new targets, disease progression, and systems pharmacology, indicating an overall shift from basic experiments toward application-oriented research.

The series of plots displayed here represents the evolution of research topics over time, from 2000 to 2024 (Figure 5C), showing a clear transition from relatively scattered and weakly connected themes in the early 2000s to progressively larger, more interconnected clusters over time. From 2006 onward, topics began to coalesce and overlap more frequently, and by 2011–2015, the clustering and cross-topic interaction became pronounced, reflecting increasing interdisciplinarity across NDD mechanisms, experimental models, and bioactive compounds. After 2016, topic overlap further intensified, with strong convergence around AD/PD-related pathways and translational efforts, including oxidative stress, neuroinflammation, proteostasis, molecular docking, and therapeutic candidate development. By 2021–2024, the landscape appears highly integrated, indicating mature cross-disciplinary research that increasingly combines molecular biology, pharmacology, and computational approaches to address complex NDD challenges.

### 4.4. Emerging Topics

**Figure 6:**
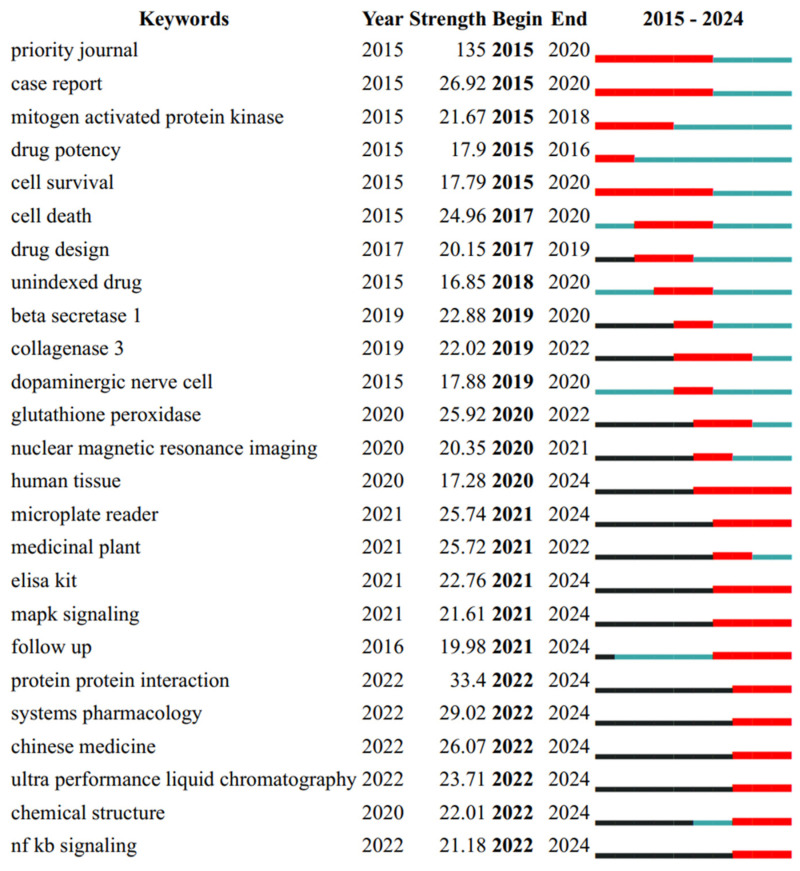
Emerging trends in polyphenol–NDD research (2021–2024). Recent/emerging keywords/topics (2021–2024), highlighting rapidly growing themes related to translation, delivery/bioavailability, and clinical endpoints/biomarkers. The red segments denote the burst intervals; black segments indicate years before the burst, and cyan segments indicate years after the burst (non-burst periods).

The keyword time zone chart provides a detailed longitudinal perspective on the evolution of research themes from 2015 to 2024 (Figure 6). In the initial phase spanning 2015–2016, research efforts were primarily concentrated on clinical investigations evaluating drug efficacy and elucidating cellular mechanisms underlying neurodegenerative diseases, with particular emphasis on Alzheimer’s and Parkinson’s disease models. These studies predominantly explored fundamental aspects, including oxidative stress pathways, cellular survival mechanisms, and protein expression profiles using conventional laboratory methodologies. The subsequent period from 2017 to 2019 witnessed a significant methodological shift toward more sophisticated approaches, with increasing emphasis on computer-aided drug design, molecular docking techniques for target identification, and expanded clinical trial initiatives. This phase also saw growing scientific interest in screening neuroprotective compounds and investigating neuroimmune interactions, reflecting an expanding understanding of disease pathogenesis. The post-2017 rise in clinical evaluation may reflect increasing translational pressure and attention to bioavailability/PK constraints, although heterogeneity in formulations, doses, and endpoints warrants cautious interpretation. Between 2020 and 2021, the research landscape became increasingly diversified, incorporating novel dimensions such as biomarker discovery and validation protocols, comprehensive pharmacokinetic assessments of drug bioavailability, and the implementation of advanced imaging technologies, including nuclear magnetic resonance spectroscopy. These developments occurred in parallel with the adoption of systems pharmacology frameworks and more refined biochemical analytical methods. The most recent research period from 2022 to 2024 demonstrates three prominent and interconnected trends: the systematic integration of traditional Chinese medicine principles into modern therapeutic development, widespread adoption of automated microplate reader technologies for high-throughput screening, and the standardization of biomarker validation protocols. These developments may indicate an emerging shift toward more integrative and application-oriented research, although the extent of this shift varies across subfields.

## 5. Clinically Investigated Polyphenols

### 5.1. Clinically Investigated Interventions Across Major NDDs

Clinical studies and human-oriented investigations have evaluated polyphenols as pure compounds, standardized extracts, or composite mixtures, with Alzheimer’s disease (AD) being the most frequently studied condition (Table 2). In AD and cognitive impairment settings, repeatedly investigated candidates include resveratrol, silymarin, chlorogenic acid, and epigallocatechin gallate (EGCG), alongside flavonoid-rich interventions such as isoflavones, genistein, and catechins; commonly studied extracts include Ginkgo biloba, olive polyphenols, and cocoa flavanols, while composite dietary mixtures (e.g., blueberry/cherry products, polyphenol-rich juices, muscadine wine, and the MIND dietary pattern) have been assessed for their potential to mitigate cognitive decline. Beyond AD, clinical or translational signals have also been explored in Parkinson’s disease (PD) and multiple system atrophy, where EGCG, curcumin, and silymarin are frequently studied, together with extracts such as green tea polyphenols and licorice polyphenol-rich preparations, and mixtures such as flavonoid-rich cocoa and saffron–chamomile formulations. For ALS and multiple sclerosis, studies have examined silymarin, EGCG, nanocurcumin, and pterostilbene, and in some cases composite formulations such as liposomal polyphenols. More limited clinical exploration exists for Huntington’s disease (e.g., EGCG and resveratrol) and frontotemporal dementia (e.g., luteolin), highlighting both emerging interest and substantial evidence gaps across less-studied NDDs.

### 5.2. Mechanistic Rationale Supporting Translational Signals

Considerable progress has been made in exploring how polyphenols may modulate biological processes implicated in NDDs, largely based on preclinical and mechanistic studies. Across experimental models, polyphenols have been reported to influence oxidative stress, neuroinflammation, intracellular signaling, and proteostasis, and some compounds have shown activity against pathological protein aggregation (e.g., Aβ/tau or α-synuclein) in model systems. For example, flavonoids (e.g., EGCG) and phenolic acids have been associated with changes in antioxidant defenses and neurotrophic signaling (e.g., BDNF/TrκB-related pathways) in preclinical settings. Non-flavonoid polyphenols such as resveratrol have also been studied for their potential to modulate amyloid-related and synuclein-related pathways, although the relevance of these findings to human disease progression remains uncertain. From a mechanistic perspective, reported effects include modulation of redox balance, inflammatory signaling (e.g., NF-κB-related pathways), and autophagy- or proteostasis-related markers, as well as possible epigenetic regulation in specific experimental contexts. Importantly, these mechanistic findings do not necessarily translate into clinically meaningful outcomes due to factors such as bioavailability, dosing, and heterogeneity across study designs [32].

Clinical and epidemiological studies have explored polyphenol-related interventions, but the overall evidence remains mixed and heterogeneous. EGCG and other polyphenols have been evaluated in human studies across selected NDD contexts [10], and standardized extracts such as Ginkgo biloba and olive polyphenols have been investigated for cognitive outcomes in mild cognitive impairment or dementia-related settings, with some studies reporting modest or context-dependent effects [13]. Observational links between polyphenol-rich dietary patterns (e.g., Mediterranean-style diets) and cognitive outcomes provide supportive but non-causal evidence. Composite polyphenol preparations and whole-food interventions may offer additive effects; however, further well-designed trials are required to clarify efficacy, optimal formulations, and target populations.

### 5.3. Nutritional Applications and Implementation Challenges

From a nutrition and food-health perspective, polyphenols can be delivered through whole-food patterns, functional foods, and nutraceutical formulations, potentially leveraging synergistic effects when consumed as composite mixtures. Epidemiological observations (e.g., polyphenol-rich dietary patterns such as the Mediterranean diet) support continued investigation of nutritional approaches, while clinically investigated interventions illustrate which compounds and preparations have progressed beyond preclinical studies. However, translation remains constrained by several persistent barriers: low and variable bioavailability, rapid metabolism, and limited brain exposure; heterogeneity in formulations, doses, and endpoints across clinical trials; and insufficient standardization and biomarker integration. Current strategies to address these issues include nanodelivery systems (e.g., liposomes and polymer nanoparticles) to enhance stability and blood–brain barrier penetration [77,78], structural modifications to improve metabolic stability [79,80], and bioenhancers (e.g., piperine) to prolong systemic exposure [81]. Inter-individual variability (microbiome and genetic factors) further motivates future work within precision nutrition frameworks [82,83]. Overall, the most actionable next steps are to prioritize standardized reporting of polyphenol composition and exposure, adopt harmonized clinical endpoints and biomarker panels, and conduct adequately powered, longer-term trials to clarify which polyphenol interventions are most promising for specific NDD contexts.

## 6. Conclusions and Future Perspectives

As a bibliometric scoping review, our analyses primarily characterize publication patterns and thematic structures; mechanistic and clinical implications are discussed cautiously and separately from bibliometric signals. By integrating bibliometric mapping of 15,073 records (1940–2024) with a targeted mechanistic evidence synthesis, this study provides a structured overview of how the polyphenol–NDD field has developed and where it is heading. Bibliometric results indicate (i) a marked acceleration of publications after 2000; (ii) geographic leadership concentrated in China and the United States; (iii) a strongly Alzheimer’s disease-centered distribution across polyphenol classes; and (iv) persistent hotspots and converging themes spanning oxidative stress/neuroprotection, neuroinflammation, cognition/aging, and animal models, with more recent growth in bioavailability, delivery technologies, and computational discovery. Complementing these patterns, the mechanistic synthesis highlights recurrent biological modules frequently investigated across representative polyphenols, including oxidative stress control, neuroinflammation modulation, proteostasis (Aβ/tau and α-synuclein), mitochondrial dysfunction, and cholinergic regulation (Section 3). However, the available clinical literature remains uneven and heterogeneous. While certain interventions (e.g., EGCG, Ginkgo biloba extracts, resveratrol, and polyphenol-rich mixtures) have been evaluated in humans, variability in formulations, exposure, endpoints, and follow-up limits the strength and generalizability of conclusions.

Future studies should prioritize (i) under-represented diseases and mechanisms identified by the mapping (e.g., prion disease, LBD, and FTD); (ii) improved standardization and reporting of polyphenol interventions (composition, bioavailability, and exposure); (iii) harmonized clinical endpoints and biomarker-informed designs; and (iv) evaluation of delivery strategies and precision-nutrition approaches in adequately powered trials. These steps are needed to better align mechanistic plausibility, bibliometric prominence, and clinically actionable evidence.

## Figures and Tables

**Table 1:** Research topics in polyphenol–neurodegeneration studies (KeyBERT and MMR).

Topic	KeyBERT			MMR		
0	antioxidant	bioactive	phytochemical	extracts	antioxidant	plant
2	alzheimer disease	neurodegenerative	dementia	alzheimer disease	disease	memory
3	beta amyloid	Pathogenesis alzheimer	amyloid precursor	beta amyloid	beta 42	alzheimer disease
4	diagnosed	patients	clinical	patients	drug	clinical
5	neuroprotective effects	oxidative stress induced	oxidative stress	oxidative stress	h2o2	ros
6	anti-alzheimer	acetylcholinesterase	acetylcholine	ache	ic50	acetylcholinesterase
8	synthesized compounds	butyrylcholinesterase	compounds	synthesized	ache	compounds
9	prion diseases	prion protein prp	prion protein	resveratrol	prion	prp
11	flavonoid	quercetin	flavonols	quercetin	flavonoids	effects
12	PD neurodegenerative disorder	disorder characterized	pd progressive	parkinson disease pd	dopaminergic	dopaminergic neurons
13	anti-neuroinflammatory	neuroinflammatory	microglia activation	microglia	microglial	neuroinflammation
14	antioxidants	functional foods	antioxidant	polyphenols	health	food
15	dopaminergic neurons	dopaminergic	pd mice	pd	mptp	parkinson
16	cerebral cortex	hippocampus	scopolamine	memory	scopolamine	learning
17	anti-aging	aging associated	risk factor neurodegenerative	aging	age related	lifespan
18	amyloidogenic proteins	amyloid related	anti-amyloidogenic	amyloid	fibrils	formation
19	effects of green tea	effects tea	tea polyphenol epigallocatechin	tea	egcg	green tea
20	cause dementia	dementia alzheimer	patients dementia	dementia	alzheimer	vad
21	cognitive decline	mild cognitive impairment	cognitive impairment	cognitive	decline	older
22	treatment of alzheimer disease	Chinese medicine	pharmacology	targets	ingredients	traditional Chinese
23	brain drug delivery	blood–brain barrier	brain targeting	drug delivery	nanoparticles	blood–brain barrier
24	amyotrophic lateral sclerosis	als fatal neurodegenerative	lateral sclerosis als	als	sod1	amyotrophic lateral sclerosis
25	alzheimer disease tauopathies	associated protein tau	inhibit tau aggregation	tau	tau aggregation	tauopathies
26	alpha synuclein oligomers	alpha synuclein	protein alpha synuclein	synuclein	aggregation	fibrillation
27	effects estrogen	selective estrogen	estrogen deficiency	estrogen	Postmenopausal women	genistein
28	monoamine oxidase mao	oxidase mao inhibitors	mao inhibitory activity	mao	mao inhibitors	monoamine
29	role mitochondrial dysfunction	mitophagy mitochondrial	mitophagy mitochondrial biogenesis	mitochondrial	mitochondrial dysfunction	biogenesis
30	depression	glutamate-induced neurotoxicity	neuroprotective	glutamate induced	excitotoxicity	depression
31	curcumin related	curcuminoid	efficacy curcumin	curcumin	curcuma	curcuminoids
0	antioxidant	bioactive	phytochemical	extracts	antioxidant	plant
2	alzheimer disease	neurodegenerative	dementia	alzheimer disease	disease	memory

**Table 2:** Clinically validated polyphenolic interventions for major neurodegenerative diseases.

Disease Characteristics	Pure Compounds:	Extracts:	Composite Mixtures:
Alzheimer’s disease cognitive impairment	Resveratrol [11,12,32,33,34] Silymarin [35,36] Chlorogenic Acid [37] Isoflavone [38] Sesamin [39] Genistein [40] Catechins [41,42] Epigallocatechin Gallate (EGCG) [43] Anthocyanin [44,45,46]	Ginkgo Biloba Extract [13,47,48] Olive Polyphenols Extract [49] Cocoa Flavanols [50]	Grape [51] Blueberry [52,53] Cherries [54] Polyphenols-Rich Fruit Juice [55,56,57] Muscadine Wine [58] MIND Food [59]
Parkinson’s disease multiple system atrophy	EGCG [10,60,61] Curcumin [62]	Polyphenol-rich extract of licorice [63]	Flavonoid-rich Pure Cocoa [64] Saffron and Chamomile [65]
Amyotrophic lateral sclerosis multiple sclerosis	Silymarin [66] EGCG [67,68] Nanocurcumin [69] Pterostilbene [70,71]		Liposomed Polyphenols [72]
Huntington’s syndrome	EGCG [73,74] Resveratrol [75]		
Frontotemporal dementia	Luteolin [76]		

## Data Availability

Primary data. All data supporting the findings of this study were obtained from publicly accessible bibliographic databases: PubMed (https://pubmed.ncbi.nlm.nih.gov/ (accessed on 5 January 2025)), Embase (https://www.embase.com/ (accessed on 2 January 2025)), Cochrane Library (https://www.cochranelibrary.com/ (accessed on 1 January 2025)), and Web of Science Core Collection (https://www.webofscience.com/ (accessed on 3 January 2025)). Searches covered records up to 31 December 2024 and were conducted in accordance with each provider’s access and usage policies. The full database-specific search strategies (including keywords, subject headings, and Boolean operators) are provided in Supplementary File S1. Processed/derived data. The consolidated, de-duplicated, and standardized metadata underlying the bibliometric analyses (e.g., title, authors and affiliations, year, keywords, and abstracts where available) and the analysis-ready outputs used for Figure 3, Figure 4, Figure 5 and Figure 6 and Table 1 (e.g., keyword co-occurrence networks, clustering/topic-model matrices) are available from the corresponding author upon reasonable request. Redistribution of source records from licensed services (e.g., Embase and Web of Science) may be restricted by provider licenses; any sharing will comply with those terms. Additional datasets. Any additional datasets generated during this study (e.g., intermediate files created during data cleaning and de-duplication) are available from the corresponding author upon request. Code and analytical workflows. Custom scripts and workflows for bibliometric processing, clustering, and topic modeling are available from the corresponding author upon reasonable request. Bibliometric analyses used Clarivate’s Derwent Data Analyzer version 12.6 (institutional subscription required), with complementary visualizations performed in VOSviewer version 1.6.10 and CiteSpace 6.3 R1. Topic modeling employed Python implementations of BERTopic. Requests for data or code should be directed to the corresponding author.

## Supplementary Materials

The following supporting information can be downloaded at: https://www.mdpi.com/article/10.3390/nu18040602/s1, Supplementary File S1: Complete database-specific executable search strings (including controlled-vocabulary and free-text terms, Boolean logic, field tags, limits/filters) and retrieved record counts for PubMed, Embase, Web of Science Core Collection, and the Cochrane Library; Supplementary File S2: Python scripts used to convert EndNote exports into a Derwent Data Analyzer (DDA)-readable schema and to standardize bibliographic fields.

## Abbreviations

The following abbreviations are used in this manuscript:

NDDs	Neurodegenerative diseases
AD	Alzheimer’s disease
PD	Parkinson’s disease
ALS	Amyotrophic lateral sclerosis
FTD	Frontotemporal dementia
LBD	Lewy body dementia
HS	Huntington’s syndrome
EGCG	Epigallocatechin gallate
MCI	Mild cognitive impairment
PRISMA-ScR	Preferred Reporting Items for Systematic Reviews and Meta-Analyses extension for Scoping Reviews
WoS	Web of Science
MeSH	Medical Subject Headings
CENTRAL	Cochrane Central Register of Controlled Trials
DDA	Derwent Data Analyzer
ISSN	International Standard Serial Number
PMID	PubMed Identifier
DOI	Digital Object Identifier
BERTopic	BERTopic (BERT-based Topic Modeling)
