# Supplementary material for: Polyphenols and Neurodegenerative Diseases: Knowledge-Mining Insights, Mechanistic Evidence, and Emerging Nutritional Applications
Source: Nutrients. 2026 Feb 12;18(4):602. doi: 10.3390/nu18040602 (PMC12943002; doi:10.3390/nu18040602)
Supplement: Supplementary file 1 [file nutrients-18-00602-s001.zip › Supplementary File S2/Derwent Data Analyzer v12.pdf]

Derwent Data Analyzer v12.6 Release Notes

Online Quick Reference Guides

https://thevantagepoint.com/help/DDA/12/12_6/zh/topics/thomsoninnovation.htm

We are pleased to announce the release of an update for Derwent Data Analyzer. Version 12.6.32704 adds several performance enhancements and stability improvements to the DDA suite. The highlights are outlined below.

Downloading and installing DDAv12.6

Details on upgrading from an earlier version can be found on our website in the **upgrade FAQ**.

Version 12.6.32704 Release Notes (March 2025)

Import

- Improved field history events for API Import and Import more fields
- Import filters can add hyperlinks to fields.
- Import .ZIP files downloaded from data providers without unzipping them first.
- Improvements to API Import to handler a wider range of available APIs.

Refine

- Enhanced UI for conflicts when merging thesauri. Allows you to see all changes at once to make quicker decisions.
- Added Capability to create Complement of Selected Groups
- Add further processing command to create readable hyperlinks to patent records.
- Automatically select source field for API query for some APIs
- New datasets now do not clear "Omit Record" flags if you choose to include them in your new dataset.
- Fixed bug where new dataset operations would lose category/sub-category relationship.
- Fixed bug running thesauri on compound fields.
- Fixed bug where some hotkeys wouldn't work in list cleanup confirmation.
- Fixed error when Combining duplicate records with some records marked for omission.

Analyze

- Updated Concept Grid algorithm for better sub-categories and smaller file sizes.

- Export and Import Multi-level record classification scheme now handles sub categories properly.

Reports

- Profile Table: Allow column re-ordering in Template Manager. Change column order in real time without having to rebuild the table.
- Add Play/Pause button to loop through Scatter Plot changes over time.
- Add Dot-paths to track Scatter Plot changes over time.
- Export Image replaced with internal tool to improve reliability.
- Store locations of dragged nodes in Maps.
- Fixed bug where Classifications and Notes would not export in fielded record export.
- Fixed bug where bubble chart would fail on some malformed dates.
- Allow Bubble Chart axis labels to display at an angle and wrap for better long label handling.
- Fixed bug in Matrix Viewer for Parent Scope matrices.
- Improved compatibility when exporting Profile Tables to older versions of Excel.
- Fixed bug in Word Cloud where some terms would appear off screen.

Autosave and File Efficiency

- Massive improvement for saving files with a lot of browser sheets
- Improve buttons for autosave recover dialog to make it more clear what the available actions are and enhanced summary of autosaved merges to make it more user friendly.
- Compress data sent to browsers to improve storage efficiency and load times

Miscellaneous

- Added controls to adjust font for the entire interface
- Improved sizing for messages to increase visibility without resizing
- Added Fuzzy files to update dialog.
- Updated chromium from 116 to 128 to improve memory and other resource allocation

- Updated libxl and other underlying libraries
- Fixed search in Chinese/Japanese Web Help

Version 12.5.31507 Release Notes (March 2024)

Autosave

- AutoSave! DDA will now save changes made to a file in a temporary file in the event of a system crash between manual saves. When reloading the file the option to merge unsaved changes into the file will be given.

Field History

- View a field's history (where it came from) by right-clicking it on the Summary sheet and selecting "View Field History"
- Track changes and events for each process used to create a field
- Add notes for any event to help you remember the specifics
- Export the entire history out to Excel to produce internal process documentation for future analyses

Refine

List Cleanup

- Improvements to List Cleanup with Thesaurus workflow when multiple matches are detected.
- Save Position of Confirmation window and Cleanup Detail Windows
- Save Detail Window fields during List Cleanup Confirm based on ruleset selected. i.e. Organization Names will pick separate fields in the Detail Windows than Person Names.
- Fixed bug in sorting list cleanup sub-items
- Add Horizontal Scroll to List Cleanup Confirm
- Add capability to zoom text in List Cleanup Confirm Window
- Editable hotkeys in the List Cleanup Confirm Dialog. Setup your own keyboard shortcuts for things like google search.
- Filter Cleanup Confirm window by count of terms in sets with "Sets>1"
- Additional filtering options for List Cleanup Confirm to see Combined by

Thesaurus, Custom Thesaurus Items, Cloned Items

Other Refine

- Speed up creation of Key Field
- Update IPC Short Label thesaurus for 2023
- Thesaurus editor - Speed improvements. View matches in real time.
- New Further Processing commands to count Words and Characters.
- Fixed bug where Further Processing would not work on compound fields.
- Fields created by Further Processing commands now keep the original field's groups.
- My Keywords - remember the last viewed list when re-opening.

Analyze

Multi-level Record Classification

- Add another tier to your Record Classification hierarchy with Sub-categories
- Records assigned to Sub-categories will automatically be assigned to its parent category.

Title and Detail Windows

- View Groups or Groups of Items in Detail Windows
- Detail Window Add search engines to right click menu
- CTRL + C is now supported in Title and Detail windows for copying data.
- Add option to sort title view by status: Classified, Omit, Note, in addition to Alpha

Report

Profile Table (Formerly Super Profile)

- Create fully customizable reports on the fly with Profile Tables
- Select items to profile and add or remove columns as you see fit to generate the report you need
- Save templates to generate profiles on future datasets to save time and effort.

World Map

- Show Record Counts on the World Map

Miscellaneous

- Improved handling for corrupt data; Aggressively identify and remove problematic fields with warnings to save the underlying file. Re-import or run processes again to create the field(s) again.
- Massive speed up of Sort Ungrouped list operation.
- Browser sheets can now handle larger datasets.
- Speed up calculation of auto-correlation matrices.
- Fixed bug where changes to visualizations wouldn't save.

Version 12.1.30406 Release Notes (April 2023)

Import

- Added Import Filter command to Read Link Data to allow importing an embedded URL from an Excel table.
- Fixed crash when canceling an import from API call.
- Improve error handling when importing from API
- Improve HTML protection in import

Refine

- Add or Edit hyperlinks on list items via context menu
- Edit Item Text allows paste without overwriting existing text.
- Fixed bug when trying to rename a My Keyword list.
- Terms by Year enhancements to improve performance and eliminate errors.
- Fixed bug in List Cleanup not matching stems properly.
- Sort groups in Dialogs based on sort order in List View.

Analyze

- Fix bug when selecting main label in Concept Grid.
- Fixed render issue when attempting to load inline links within a block of text in record views.
- Fixed bug running Cluster Records in non-US locales.

- Fixed bug selecting records in Cluster Records.

Report

- Fixed axis label in Scatter Plot.
- Fixed font styles not being applied properly after initialization.
- Fixed bugs in Export Image PNG export introduced by chrome 111 where canvas size defaults to 800x600 and some elements were not properly rendered.

Miscellaneous

- Fixed dialogs losing focus after autosize

Version 12.1 Release Notes (released December 2022)

Import

- Added Dataset Identifier field to default import filters to differentiate between “old” and “new” records when combining datasets.

Refine

- Fixed issue where process fields were being added to the record view.
- Fixed bug in Remove Duplicates where selecting records by date wasn’t ordered properly.
- Fixed bug in Remove Duplicates where matching criteria was dependent on the order they were selected.
- Fixed issues with child fields and further processing commands breaking child history or improperly assigning processed fields as active children.
- Fixed bug in Cleanup Confirm window improperly highlighting terms as matched by thesaurus.
- Fixed bug resuming saved list cleanup when using a thesaurus during cleanup.
- Improvements to Refine NLP to remove more “junk” terms.

Analyze.

- Added field selection dialog for Concept Grid to limit to only fields that make sense for the algorithm.
- Fixed bug in Pivot Tool to properly count record counts for multi-valued fields when filtering.

- When importing categories in the Record Classification dialog, users will now be prompted to automatically assign records to those categories if they come from a field within that dataset.
- Allow user to bias terms as “most important” for the auto-classifier by assigning a keyword to a classification in the auto-classifier settings dialog.
- In Smart Trainer, prioritize English Only records first for training.

Report

- Add Record Classification and Record notes to fields shared during “Record Share”.

Miscellaneous

- Fixed bugs and stability issues.
- Updated Context Sensitive Help (F1) for many dialogs and visualizations.
- Fixed alpha sorting for field names on some dialogs.
- After resizing, dialogs reposition to the center of the screen so buttons are visible.
- Updates to Chromium and Stingray Libraries.
- Force DDA window to the front after completing a script.

Version 12 Release Notes (released September 2022)

New capabilities for Find Records

- Search for records based on group membership
- Search for records based on record note text

Refine

Add To Group (new Dialog)

Accessed via Right click-> Add to Group in List/Detail/Matrix views and in Find -> Add to Group. This change adds the ability to type to filter groups, which is especially useful when looking for a specific group out of 50+ groups.

Combine Duplicate Records (new look)

More intuitive interface allows users to quickly identify which fields to match on.

Remove Duplicate Records (new look – new capability)

The new interface allows users to select multiple criteria for choosing which record to keep when removing duplicates. These criteria, which can be layered as a filter, include record length, dates, values, group membership, language, as well as specific terms. This flexible, user-defined filter enables customers to precisely specify which record to keep when removing duplicates

Split items into multiple items in List Cleanup Confirm

In List Cleanup Confirm an item can now be split into two. This is useful when an item contains two different items in one. E.g. “Georgia Tech and Univ of Georgia” can be cloned and added to both the “Georgia Tech” and “Univ of Georgia” sets separately, preserving record counts.

IPC-WIPO Green Codes

Adds a definition file for converting IPC Codes to WIPO's environmentally friendly technology categories.

Analyze

Network Metrics

Users can analyze a set of individuals (as well as other fields) using Network Metrics. The feature allows the user to select which field to use for the network calculation. The feature then calculates Betweenness Centrality, Closeness Centrality, and Degree Centrality. The feature also calculates a composite Keystone Indicator consisting of all three measures which ranges from 0 to 1. The higher the Keystone Indicator, the more networked an individual is within the search space.

- Betweenness Centrality – how often a node lies along the path between other nodes. (i.e. a lot of information flows through that node)
- Closeness Centrality – total distance to all other nodes
- Degree Centrality – how many connections does a node in the network have?
- Keystone - Harmonic Mean of the three centrality measures and the number of records.

Smart Trainer – add fields to Smart Trainer View

Enables user to select and display non-training fields in the Smart Trainer view to help provide context during training

Sankey Chart

Create a flow chart with up to 5 fields showing the cooccurrence between the fields.

Records will cascade down from left to right. In other words, data in the columns will not contain any records that were not in set of records in the column to the left of it.

Limited to a max of 5 fields.

Clicking the link between two nodes in the chart will show the shared records between those two items in the data.

Create Concept Tree

This algorithmic approach to clustering concepts uses NLP phrases as an input. The algorithm then conducts a series of optimized clustering calculations to divide the topic into major areas. The algorithm then divides the major areas into minor areas. Finally, the algorithm visualizes the output, names the areas, and color codes overlapping minor areas.

Report

Add Third Field to Bubble Chart (Bubble Pie)

Users can add additional data to DDA Bubble Chart by including a Pie Chart within each bubble

Export to Tableau and PowerBI

- Users can export content out of DDA to import into PowerBI.
- Users can export content out of DDA to import into Tableau.

Share Record and Email

Customers can use the flexible Record View layout to customize how records are displayed including which fields are shown (including images) and what order they are shown in, as well as highlighting. A record can then be shared with others as a PDF**.**
